# Effects of Chronic Exercise on Endothelial Progenitor Cells and Microparticles in Professional Runners

**DOI:** 10.5935/abc.20170022

**Published:** 2017-03

**Authors:** Célia Regina de Oliveira Bittencourt, Maria Cristina de Oliveira Izar, Carolina Nunes França, Valdir Lauro Schwerz, Rui Manuel dos Santos Póvoa, Francisco Antonio Helfenstein Fonseca

**Affiliations:** 1 Universidade Federal de São Paulo (UNIFESP), São Paulo, SP - Brazil; 2 Universidade de Santo Amaro, São Paulo, SP - Brazil

**Keywords:** Endothelial Progenitor Cells, Biomarkers, Athletes, Sports, Running

## Abstract

**Background:**

The effects of chronic exposure to exercise training on vascular biomarkers have been poorly explored.

**Objective:**

Our study aimed to compare the amounts of endothelial progenitor cells (EPCs), and endothelial (EMP) and platelet (PMP) microparticles between professional runners and healthy controls.

**Methods:**

Twenty-five half-marathon runners and 24 age- and gender-matched healthy controls were included in the study. EPCs (CD34+/KDR+, CD133+/KDR+, and CD34+/CD133+), EMP (CD51+) and PMP (CD42+/CD31+) were quantified by flow-cytometry. All blood samples were obtained after 12 h of fasting and the athletes were encouraged to perform their routine exercises on the day before.

**Results:**

As compared with controls, the CD34+/KDR+ EPCs (p=0.038) and CD133+/KDR+ EPCs (p=0.018) were increased, whereas CD34+/CD133+ EPCs were not different (p=0.51) in athletes. In addition, there was no difference in MPs levels between the groups.

**Conclusion:**

Chronic exposure to exercise in professional runners was associated with higher percentage of EPCs. Taking into account the similar number of MPs in athletes and controls, the study suggests a favorable effect of exercise on these vascular biomarkers.

## Introduction

An appropriate number of circulating endothelial progenitor cells (EPCs) seems related with the maintenance of vascular homeostasis.^1,2^ In fact, decreased number of EPCs has been associated with cardiovascular risk factors, cardiovascular mortality, and recurrent cardiovascular events in subjects with coronary heart disease,^3,4^ despite some controversies regarding the measurement, characterization, origin and destiny of such cells.^5,6^

Microparticles (MPs) are small cell-derived anucleoid phospholipid particles (100-1000 nm) that can be identified by their origin from endothelium (EMP), platelets (PMP) or many other cells. Increased number of EMPs has been linked with endothelial injury or endothelial dysfunction.^7,8^ Interestingly, PMPs, initially considered markers of thrombosis, are now considered relevant for some transcriptional signaling, for the interaction with monocytes and activation of inflammatory responses.^9^

Regular exercise has been widely recommended for prevention of cardiovascular disease, but information regarding the effects of chronic and intense exposure to exercise on these vascular biomarkers is scarce.^10,11^ Thus, the objective of this study was to evaluate the effects of chronic exercise in professional runners on EPCs and MPs.

## Methods

### Study population

Professional half-marathon runners (n=25) and age and gender-matched controls (n=24) without known cardiovascular diseases were prospectively included. Subjects with cardiovascular risk factors such as hypertension, diabetes, obesity, smoking, or hypercholesterolemia were excluded. The local ethics committee approved the study (# 1808/08) and all participants have signed the informed consent prior to their inclusion in the study protocol.

#### Laboratory analysis

Blood samples were obtained after 12 hours of fasting and the analyses were performed at the central laboratory of our university. All athletes were allowed to maintain their daily exercises program even on the day before blood sample collection. The athletes had very similar exercise training programs, corresponding to two long-distance running sessions every day, 15 km in the morning and 10 km in the afternoon, and intensive training (100-1,000 meter shots, repeated many times)twice a week, on Tuesday and Thursday mornings. All blood samples were collected on Thursdays, before exercise.

Measurements of EPCs and MPs were performed as previously reported, using fresh blood samples in EDTA containing tubes.^12-15^ For determination of EPCs, a minimum of 500,000 events was acquired by flow-cytometry (FACSCalibur, BD Biosciences, USA). Fluorescently labeled mouse anti-human antibodies were used for EPCs (CD34 FITC, BD Biosciences, USA; CD133 APC, Miltenyi Biotec, USA; KDR PE, R&D Systems, USA), PMPs (CD42 FITC and CD31 PE, BD Biosciences, USA) and EMPs (CD51 FITC, BD Biosciences). Disposable containers (BD Biosciences) were used to quantify the number of microparticles *per* microliter of platelet-poor plasma (PPP).

#### Statistical analysis

Results are presented as mean ± standard deviation (SD) or by median and interquartile range (IQR), for normal or non-Gaussian distributions, respectively. Categorical variables were compared by Pearson's Chi-square test. Kolmogorov-Smirnov and Shapiro-Wilk tests were used to assess normality of continuous variables. Between-group comparisons of continuous variables were made by unpaired t-test or Mann-Whitney test, when appropriate. Spearman's rank correlation test was used to evaluate correlations of EPCs and MPs with variables of ergospirometry. All analyses were performed using SPSS 17.0 for Windows (SPSS, Inc., Chicago, IL) and significance was set at p<0.05.

## Results

All athletes reported to have exercised on the day before (22.08 ± 2.67 km, mean ± SD), and the mean time between the last exercise session and blood collection was 16.5 ± 2.8 hours. Male and female athletes did not differ in both distance (124±25 vs. 128±29 km per week, p=0.88, respectively, mean ± SD, unpaired t test) and time spent in training (14±4 vs. 14±7 hours per week, mean±SD, p=0.53, respectively, unpaired t test). Despite exposure to the same training regimen, male athletes reported better mean time for 10,000 meters than female athletes (32.4±2.1 vs. 37.6±1.6 min, p<0.0001, mean±SD, unpaired t test). As compared with controls, athletes had lower weight, body mass index, abdominal circumference and percentage of body fat, lower heart rate, and higher body lean mass, but similar values of systolic and diastolic blood pressure. In addition, they presented lower serum levels of total cholesterol, LDL-C and triglycerides, and higher serum levels of HDL-C than controls.

### Endothelial progenitor cells and microparticles

**Figure 1:**
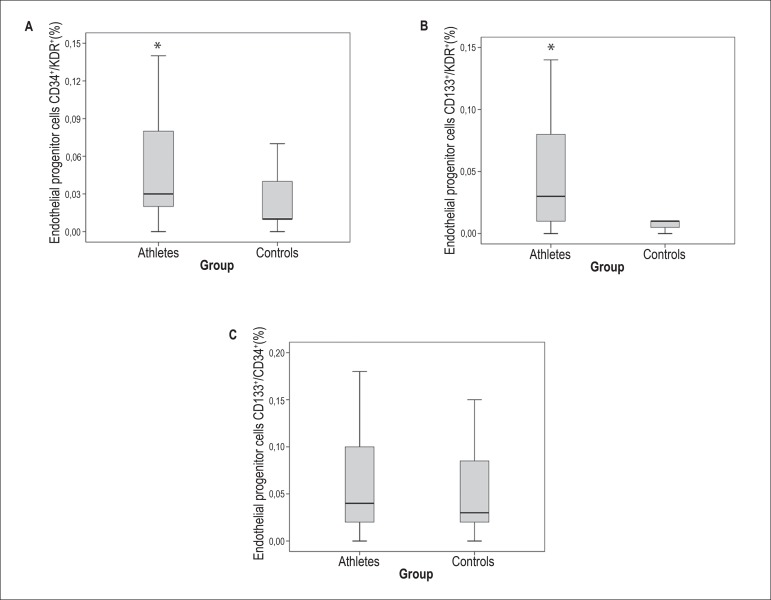
Box-plots showing the percentage of circulating endothelial progenitor cells (EPCs) determined by flow-cytometry. Higher percentage of CD34+/KDR+ EPCs (A) (p=0.038 vs. controls, Mann-Whitney U test), as well as CD133+/KDR+ EPCs (p=0.018 vs. controls, Mann-Whitney U test) (B) were found in athletes. No differences were observed between groups for CD133+/CD34+ (p=0.51) (C).

Compared to controls, the athletes presented higher percentage of two lineages of EPCs (CD34+/KDR+, and CD133+/KDR+) and similar percentage of CD34+/CD133+ cells (Figure 1).

**Figure 2:**
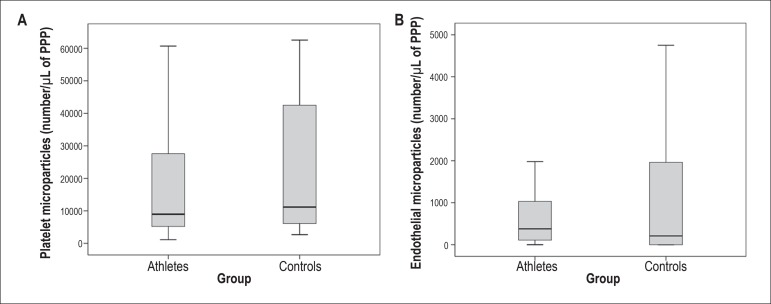
Box-plots representing the amount of circulating microparticles determined by flow-cytometry. The number of CD42+/CD31+ platelet microparticles (PMPs) (A) and CD51+ endothelial microparticles (EMPs) (B) was similar between the groups. (PMPs, p=0.695, Mann-Whitney U test; EMPs, p=0.496, Mann-Whitney U test). PPP - platelet-poor plasma.

The amount of EMPs and PMPs did not differ between the two groups (Figure 2).

No correlation between the percentage of EPCs or MPs with variables of ergospirometry was observed, including absolute and maximum rate of oxygen consumption (VO_2_max) (data not shown).

## Discussion

The present study revealed that the chronic exposure to exercise training among professional runners was associated with increased percentage of circulating EPCs without changes in the amount of EMPs or PMPs. These findings suggest that chronic exercise was not associated with endothelial cell apoptosis or thrombosis. In fact, it seemed to have a protective effect in these subjects, taking into account the observed increase in EPCs. In our athletes, blood samples were collected during their routine training program, since we wanted to evaluate EPCs and MPs in real-life context.

Several cardiovascular risk factors including diabetes,^3^ hypertension,^16^ smoking,^17^ hypercholesterolemia,^18^ and age.^19^ have been related to reduced function of circulating EPCs. Conversely, exercise has been recognized as a promise tool to increase EPCs.^20,21^ Early experimental and clinical studies^22,23^ reported increased number of EPCs after regular exercise, although the effects of exercise on EPCs seemed to be influenced by training regimen, age of subjects, and concomitant presence of cardiovascular disease, such as coronary heart disease or heart failure.^20^

Circulating EMPs have been linked to several stimuli, including the transcription of interleukins, chemokines and chemoattractants mediated by activation of nuclear factor-κB (NF-κB), and associated with oxidative stress.^8,24^ All these conditions have been long associated with classical cardiovascular risk factors, but more recently, new biological effects mediated by EMPs have been considered, including transport of mRNAs, microRNAs and other active molecules of physiologic relevance for angiogenesis and tissue repair.^25^

Cellular activation and apoptosis are linked to release of MPs. Of special interest, the amount of PMPs has been recognized as a possible marker of thrombosis, due to their high content of phospholipids and potential pro-thrombogenic roles because of thrombin generation.^26^ Besides, high shear stress triggers platelet aggregation and release of platelet derived MPs. ^27^ In addition, circulating PMPs may carry tissue factor (TF), which can also generate thrombin and platelet activation. However, it is also true that MPs may transport some inhibitors of coagulation, such as the TF pathway inhibitor (TFPI) that can neutralize, in part, the procoagulant properties of these MPs.^28^ More recently, interesting aspects linking PMPs to the signaling of inflammatory and immune responses have been proposed, considering the potential transcriptional factors in the platelets, that include nuclear factor kappa β (NF-κKB) and peroxisome proliferator-activated receptor gamma (PPARγ).^29^

In our study, we found increased percentage of EPCs in athletes and similar number of EMPs and PMPs in comparison with healthy controls, despite the intensive training of these professional athletes. These promising findings are important because our understanding of the role of exercise on EPCs and MPs is mainly derived from acute exposure or in non-athletes.^10,11,30,31^ Intermittent and high-intensity exercise induces catecholamine release and decreases highly differentiated T cells, but does not increase the amount of EPCs compared with continuous exercise^33^. In other article, despite increase in white blood cells count, the amount of EPCs observed in advanced-aged marathon runners was not modified when collected in the early period after the race.^33^

In addition, among other biochemical variables, C-reactive protein levels were lower in athletes than in controls, and creatine phosphokinase levels modestly increased, even with the routine training on the day before blood sample collection, reinforcing protective properties of high-performance exercise.

### Study limitations

Although this was a cross-sectional, case-control study, our results cannot be considered as hypothesis generating, since we do not have baseline laboratory values of the athletes. Finally, these results are applicable to marathon runners and cannot be extrapolated to other sports.

## Conclusions

Chronic exercise was associated with a favorable increase in EPCs, without affecting circulating levels of MPs in professional runners, suggesting a positive impact of prolonged exposure to chronic exercise on these vascular biomarkers.
